# Cardiac Multi-Frequency Vibration Signal Sensor Module and Feature Extraction Method Based on Vibration Modeling

**DOI:** 10.3390/s24072235

**Published:** 2024-03-30

**Authors:** Zhixing Gao, Yuqi Wang, Kang Yu, Zhiwei Dai, Tingting Song, Jun Zhang, Chengjun Huang, Haiying Zhang, Hao Yang

**Affiliations:** 1 Institute of Microelectronics of the Chinese Academy of Sciences, Beijing 100029, China; gaozhixing@ime.ac.cn (Z.G.); wangyuqi231@mails.ucas.ac.cn (Y.W.); yukang@ime.ac.cn (K.Y.); daizhiwei@ime.ac.cn (Z.D.); zhangjun@ime.ac.cn (J.Z.); huangchengjun@ime.ac.cn (C.H.); zhanghaiying@ime.ac.cn (H.Z.); 2 University of Chinese Academy of Sciences, Beijing 100049, China

**Keywords:** ultra-low-frequency seismocardiography, seismocardiography, phonocardiography, cardiac multi-frequency vibration model, vibration sensor, 1D-CNN

## Abstract

Cardiovascular diseases pose a long-term risk to human health. This study focuses on the rich-spectrum mechanical vibrations generated during cardiac activity. By combining Fourier series theory, we propose a multi-frequency vibration model for the heart, decomposing cardiac vibration into frequency bands and establishing a systematic interpretation for detecting multi-frequency cardiac vibrations. Based on this, we develop a small multi-frequency vibration sensor module based on flexible polyvinylidene fluoride (PVDF) films, which is capable of synchronously collecting ultra-low-frequency seismocardiography (ULF-SCG), seismocardiography (SCG), and phonocardiography (PCG) signals with high sensitivity. Comparative experiments validate the sensor’s performance and we further develop an algorithm framework for feature extraction based on 1D-CNN models, achieving continuous recognition of multiple vibration features. Testing shows that the recognition coefficient of determination ($R^{2}$), mean absolute error (MAE), and root mean square error (RMSE) of the 8 features are 0.95, 2.18 ms, and 4.89 ms, respectively, with an average prediction speed of 60.18 us/point, meeting the re-quirements for online monitoring while ensuring accuracy in extracting multiple feature points. Finally, integrating the vibration model, sensor, and feature extraction algorithm, we propose a dynamic monitoring system for multi-frequency cardiac vibration, which can be applied to portable monitoring devices for daily dynamic cardiac monitoring, providing a new approach for the early diagnosis and prevention of cardiovascular diseases.

## 1. Introduction

Cardiovascular diseases pose long-term risks to human health and the trend of cardiovascular diseases affecting younger populations has become increasingly prominent in recent years [1]. Therefore, early and effective diagnosis of heart disease is extremely important. The heart possesses multimodal characteristics [2]. During an exercise cycle, the cardiac sinus node sends out electrical signals that are transmitted to the cardiomyocytes to produce contraction and diastole of the myocardium, which drives the blood to flow. This process generates various mechanical wave vibrations. Ballistocardiography (BCG, whole-body motion produced by blood ejection into a blood vessel) [3] and seismocardiography (SCG, localized vibration of the chest wall induced by a heartbeat) [4] are able to represent the process of myocardial systolic-diastolic motion. Phonocardiography (PCG, 20–100 Hz) [5] is a sound signal generated by blood flow during the opening and closing of valves. Gyrocardiography (GCG) [6] embodies information about the cardiac rotational motion in different directions. The vibrational waves described above are essential for people to recognize information about cardiac motion, reflecting structural abnormalities and altered ejection capacity of the heart. The 24-h ECG Holter reflects the daily electrical behavior of the heart but the ECG signal alone does not reflect the mechanical information described above. The realization of dynamic monitoring of cardiac mechanical vibration can expand the dimensions of cardiac monitoring [7] and the combination of electrical signals can reflect more comprehensive cardiac information.

Since the 1990s, miniaturized and lightweight mechanical vibration sensors have been increasingly utilized in portable cardiac monitoring systems. Accelerometers based on microelectromechanical systems (MEMS) technology are widely used for SCG detection [8,9] and have been extended to a variety of application scenarios, including heart rate detection [10], assessment of hemodynamic parameters such as blood pressure [11] and myocardial contractility [12], and postoperative patient monitoring [13]. MEMS-based gyroscopes are used to extract GCG [6]. An Inertial Measurement Unit (IMU) allows the simultaneous extraction of 3-axis SCG signals and 3-axis GCG signals on a single miniaturized device, obtaining more information than a single sensing method [14]. A wearable hermetically sealed high-precision vibration sensor combines the properties of an accelerometer and a contact microphone to monitor a wide range of health information induced by cardiopulmonary vibration [15]. A stretchable sensor incorporating soft gold electrodes can be attached directly to the skin to extract cardiac time intervals [11]. Deformable conductive bioelectronics are increasingly being used to monitor the electrophysiologic state of the heart to improve diagnostic accuracy [16].

Based on the extraction of various vibration waves, people have gradually established a macroscopic connection between them. There is an overlap of frequencies between the vibration waves, so they are correlated. For example, both SCG (energy concentrated at 0.5–30 Hz) and PCG (energy concentrated at 20–200 Hz) can extract information about valve closure (MC, AO, AC, and MO for SCG as well as S1 and S2 for PCG). Researchers identified the vibration corresponding to PCG using vibrocardiography (SCG and GCG) [17], extracted the PCG process from SCG [18], and annotated the features of SCG by GCG [19]. Meanwhile, the vibration wave reflects different motion information of the heart because of the various frequencies. Among the heart signals extracted from the accelerometer, the band-pass filtered signal of 18–200 Hz represents the information of opening and closing of the heart valves, while the band-pass filtered signal of 0.6–20 Hz embodies the information of the recoil force of the heart contraction [20]. In more detail, signals below 0.5 Hz characterize respiratory rhythms [21]; signals around 1–5 Hz characterize cardiac volume changes and ejection phase activity [22,23]; SCG signals around 5–30 Hz characterize the opening and closing of cardiac valves as well as the systolic and diastolic processes [22]; and PCG signals around 20–200 Hz characterize high-frequency acoustic activity. Recently, researchers have been using piezoelectric transducers to synchronously extract signals in multiple frequency bands as described above [22,23,24], thanks to the broadband nature of piezoelectric methods. Simultaneous passive sensing is another advantage of piezoelectric methods, which can generate charges without an external power source. Therefore, a miniaturized high-sensitive piezoelectric transducer module can be designed for multi-frequency vibration detection in the heart, allowing the extraction of comprehensive mechanical information about the heart.

Furthermore, dynamic monitoring of cardiac activity relies on accurate and efficient feature extraction methods and researchers are currently keen to investigate automatic annotation algorithms. For ULF-SCG below 5 Hz, the ‘findpeaks’ function (from MATLAB) can extract the locations of the feature points (peaks and troughs). For PCG above 200 Hz, the locations of S1 and S2 can be identified using the energy spectrum and envelope methods [25]. The SCG signals around 5–30 Hz cover the infrasound and audible bands and have nine feature points. Because of the weak interpretability and high variability of waveform morphology, the feature extraction of SCG has become a difficult task in research. Currently, most methods can only extract some feature points with high-frequency bands. The Gaussian mixture model approach extracted two feature points (AC and AO) with low latency [26]; researchers achieved the labeling of IM and AC of SCGs by using the envelopes of the high-frequency signals from the same accelerometer [27]; and the wavelet transform approach can locate both AO and IM points [28]. The binary classifier approach is capable of extracting multiple feature points but the introduction of integrated features makes the results dependent on the morphology of the waveform [29]; the curvature method [22] is capable of extracting multiple feature points regardless of the waveform morphology but sacrifices timeliness due to its susceptibility to interference and the need for single-cycle averaging processing to achieve high recognition accuracy. Therefore, it becomes a challenge to meet the requirements of high recognition of morphological variability robustness, low latency, and handling multiple feature points.

Recently, 1D-CNN approaches have become attractive for complex engineering because they do not require manual feature production and can directly extract “learned features” [30]. Some researchers have performed 1D to 2D conversion to apply deep CNN methods [31] but the high computational complexity makes them unsuitable for real-time operation on mobile and low-power/low-memory devices. Compact adaptive 1D-CNN can [32] operate directly on one-dimensional physiological signals with low time complexity. Early arrhythmia detection in ECG beats [33] and feature extraction of pulse waves [34] are successful 1D-CNN applications.

In this study, first of all, we described the multi-frequency vibration model of the heart. Combined with Fourier series theory, the heart vibration is represented as a synthesis of vibration waves in different frequency bands and the signal is validated using EMD decomposition. Then, we propose a sensor module based on the PVDF piezoelectric film for simultaneous acquisition of multi-frequency vibrations of the heart allowing detecting both ultra-low frequency cardiac signals (ULF-SCG) and SCG signals [22]. Besides, we further optimized the volume of the sensor and designed a separate structure. Comparative experiments verified that the sensor can also extract PCG signals simultaneously. The new sensor module achieves an output sensitivity of 40.6 V/N for the module with a mass of 2.4 g in a volume of 30 × 15 × 5 mm. Meanwhile, we designed an algorithmic framework based on 1D-CNN for feature extraction of multi-frequency vibration. We use the R-wave of ECG to truncate the vibration signals of multi-frequency bands to form many single-cycle waveforms, which are input into 1D-CNN to learn the feature point locations through training and finally output the feature point coordinates. Our study recognizes two feature points of PCG and eight feature points of SCG. Excluding the two feature points of RE and RF, the average $R^{2}$, RME, and RSME reach 0.95, 2.18 ms, and 4.89 ms, respectively, and have high recognition robustness against morphological variability. The average response time of the new method to process a single feature point is 60.18 μs which achieves low latency. Finally, combining the multi-frequency vibration model, acquisition sensor, and signal feature extraction method form a cardiac multi-frequency vibration dynamic monitoring system scheme. This scheme is a more efficient option that can be applied to portable acquisition devices for daily dynamic cardiac monitoring.

## 2. Materials and Methods

### 2.1. Multi-Frequency Vibration Model and System Framework

Cardiac motion is a quasi-periodic oscillatory motion. The heart is excited by electrical signals that produce contraction and diastole of the myocardium, which drive the blood to move, thus generating various mechanical vibrations. Assuming that cardiac activity is strictly periodic and linearly stable, the vibration of the heart at a point in the chest is a superposition of periodic oscillations and noise fluctuations:

(1) $$f_{Cardiac}=f_{T}+N$$

where $f_{Cardiac}$ denotes the vibration signal of the heart at a certain point, $f_{T}$ denotes the periodic oscillation, and N denotes the noise fluctuation. $f_{T}$ can be decomposed into vibrations of different frequency bands. The vibrations in different frequency bands originate from different cardiac events, such as diastolic contraction of the ventricles and atria, valve opening and closing processes, ejection processes, and cardiac filling processes. Cardiac events produce different periodic vibrations because of different motion characteristics transmitted to the chest wall. Fourier series theory decomposes any periodic function that satisfies certain conditions into sine and cosine waves of various frequencies. Similarly, we decompose the periodic oscillations of the heart into vibrational waves of different frequency bands as follows:

(2) $$f_{T}=a_{0}+\sum_{n=1}^{m}a_{n}f_{n}(t)$$

where $f_{n}(t)$ represents vibrational waves and different vibrational waves represent different cardiac events (the greater the n, the greater the frequency of the vibrational wave). n=1 represents the fundamental frequency component of cardiac motion, i.e., the vibrational waves caused by the lowest-frequency cardiac events, which can be physiologically interpreted as the systolic and diastolic process of the heart at 60–100 times per minute. n>1 represents the various harmonic components of cardiac motion, including high-frequency events such as valve opening and closing, systolic cardiac ejection, etc. The maximum value of n is m, indicating that the number of cardiac events is finite. $a_{0}$ represents the DC component and $a_{n}$ represents the coefficients of the vibrational waveform. $f_{n}(t)$ as a periodic function can further expand into a Fourier series:

(3) $$f_{n}(t)=b_{0}+\sum_{n=1}^{\infty }\left(b_{n}cos(2\pi n/T\cdot t)+c_{n}sin(2\pi n/T\cdot t)\right)$$

where $b_{0}$ is the DC component, $b_{n}$ and $c_{n}$ are the Fourier coefficients, and 2πn/T is the angular frequency. The minimum vibration units for periodic functions in Fourier series theory are sine and cosine waves. We set the minimum unit of vibration of the heart as different cardiac events because the interpretability is stronger. And the cardiac events can be further decomposed into sine and cosine.

**Figure 1:**
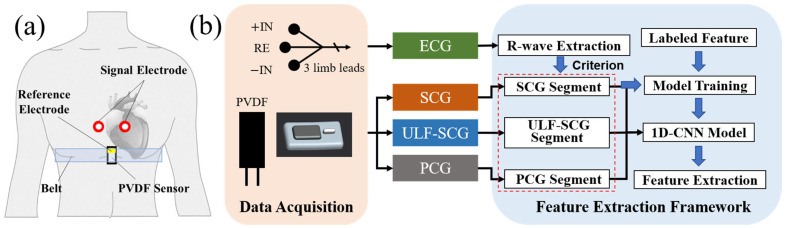
Cardiac multi-frequency vibration detection system. (**a**) Schematic diagram of the system acquisition scene. (**b**) System framework, including the sensor, a signal acquisition terminal, and feature extraction framework.

The above equations describe the multi-frequency vibration model of the heart and explains the meaning of multi-frequency vibration waves of the heart on a mathematical level. The cardiac event is the smallest unit that causes the multi-frequency vibration of the heart and is the source of the multi-frequency vibration signal at the thoracic position. The signal validation of the model is shown in Section 3.4. The model proves the importance of multi-frequency vibration signals for heart monitoring, which supports us in designing the corresponding sensors and analyzing and processing the multi-frequency signals. We established a system framework shown in Figure 1, where portability and low latency are the essential goals of the system, making it applicable to daily dynamic cardiac monitoring.

### 2.2. Sensor Design and Fabrication

This section describes the design of the PVDF-based multi-frequency sensor module and analyzes the multi-frequency signals extracted from the sensor signal.

**Figure 2:**
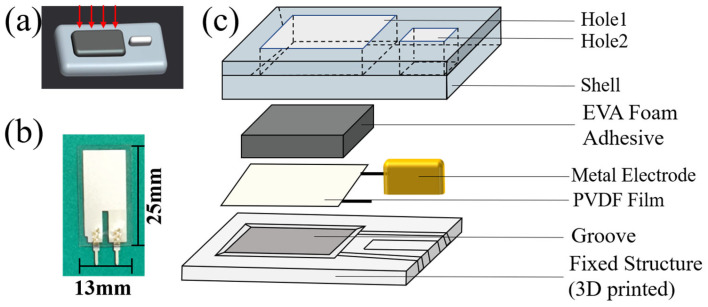
(**a**) Three-dimensional shape drawing of the multi-frequency vibration sensor, the red arrow represents the direction of pressure transfer in the chest wall; (**b**) PVDF film; (**c**) Schematic of the laminated structure of the sensor module. From bottom to top are the fixed structure, the PVDF film, the metal electrode, the EVA foam adhesive, the shell, and the two holes in the shell.

Figure 2a shows the overall structure of the multi-frequency sensor, which consists of three parts: sensing component, fixed structure, and conduction medium. The sensing component is LDT0-028K (PVDF film, TE Connectivity) with a thickness of 28 um, size of 25 mm × 13 mm, and a frequency band of 0.1–10,000 Hz, as shown in Figure 2b. The excellent wide-band characteristics make it a suitable device for recording multi-frequency vibration. In the fixation solution, we select a 3D-printed hard substrate as the fixed structure, which can transmit the force of the chest wall stably to the sensing surface. The extraction of multi-frequency vibration also requires high sensor sensitivity and PVDF sensors without a substrate have the best sensitivity because they allow sufficient epidermis deformation [11]. We designed a groove on the fixed structure to provide the best surface deformation for PVDF, enabling the module to have enough output sensitivity. The groove depth is designed at 0.35 mm to increase the vertical deformation, thereby increasing the output charge. An EVA-based foam rubber is glued on the PVDF as the conductive medium, which contacts the human chest wall to effectively conduct vibrations. In addition, to reduce the number of ECG electrodes, the reference electrode is placed on the same side of the PVDF film and designed to be a strip metal electrode, as shown in Figure 2c. We added the shell to stabilize the internal structure and dug two holes in the shell to expose the sensitive surfaces of the PVDF and metal electrodes.

In our previous study, we have demonstrated that this type of sensor can obtain ULF-SCG and SCG signals, in which ULF-SCG exhibits mapping of cardiac volume variation (the falling wave corresponds to the heart ejection phase) [22] and SCG exhibits 9 standard feature points [8,22]. In this study, we designed a separate sensor structure, shrank the sensor size, and further verified that this sensor can additionally extract PCG signals.

We perform the sensitivity test and the PCG signals comparison test in Section 3.3. The sensor achieves an output sensitivity of 40.6 V/N within a small volume of 30 × 15 × 5 mm and a mass of 2.4 g. The sensor can accurately extract the multi-frequency cardiac signals of ULF-SCG, SCG, and PCG while meeting the portable demand.

### 2.3. Acquisition Terminal Design

**Figure 3:**
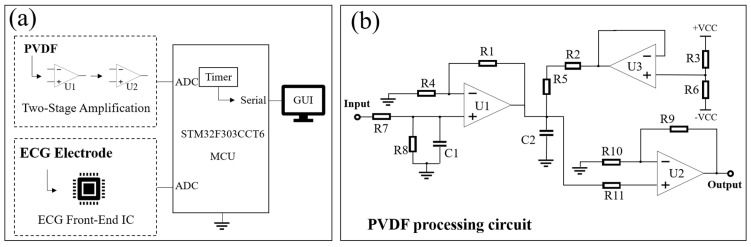
(**a**) Circuit structure diagram of the acquisition terminal, including the PVDF processing circuit, ECG analog front-end and control circuit; (**b**) Circuit diagram of the PVDF processing circuit, with the signals passing through the two-stage op-amps.

The feature extraction algorithm for cardiac multi-frequency vibration signals needs ECG as a reference. Thus, we developed a joint acquisition device to record the PVDF and ECG signals synchronously. The hardware structure is shown in Figure 3a.

The sternum is selected as the collection location of PVDF and two wires are used to deliver polarized charge to the front-end charge amplification circuit (CA3140), whose high input impedance can well capture the weak charge generated by PVDF. A total voltage gain of 29.5 dB is achieved after the two-stage amplification [22]. In addition, we used the stabilized voltage from the voltage follower as a reference to lift the output processed by the dual-supply op-amp into the input range of the ADC. The circuit structure is shown in Figure 3b.

The two processed analog signals are continuously converted using the on-chip ADC of the stm32f303cct6 MCU and the resolution is set to 12 bit. The converted digital signals are transmitted to the host computer through a serial port. The timer is set to transmit the signals every 0.5 ms so the achieved sampling rate is 2 kHz.

We adhere the ECG electrodes on the chest and use ASIC AD8232 as the analog front-end of ECG. This IC features a high common-mode rejection ratio, low power consumption, and low noise [35] and its peripheral circuit is simple. A 0.05–37 Hz band-pass filter and an amplification gain of 52 dB can be achieved by simply adjusting the resistors and capacitors.

Figure 1a shows the acquisition scene of the signal: the PVDF sensor is fixed in the sternal position by a belt and ECG signal electrodes are attached to the upper side (the ECG reference electrodes are designed on the PVDF module).

### 2.4. Feature Extraction Algorithm Based on 1D-CNN

#### 2.4.1. Feature Extraction Framework

After preprocessing, the synchronous ECG and three vibration signals were obtained. There are thirteen vibration feature points, including the WC and WT of ULF-SCG, nine feature points of SCG, and two feature points of PCG. Among them, the WT points of the ULF-SCG signal are not included in the range of feature point extraction because of their obvious features. The SCG signal has the most complexity with nine characteristic points, making feature extraction the most challenging. Among them, the AS wave is excluded from the ex-traction range due to difficulty in identification and extraction from continuous waveforms. Existing curvature methods can extract the nine characteristic points of the SCG but they require averaging over a single cycle to improve the signal-to-noise ratio (SNR), which significantly increases the delay [22]. Compact 1D-CNN provides new ideas, by using fixed-length inputs and treating feature points as labels. The feature recognition model can be obtained through sufficient training samples.

This study utilizes the ECG signal as a reference signal and employs an R-wave extraction algorithm [36] to obtain the positions of R-waves in the ECG. By truncating the three vibration signals, multiple single-cycle signals are obtained and used as input signals for the 1D-CNN model, with annotated feature points as labels of the dataset. The position of feature points can be obtained by inputting a single-period signal into the trained model to achieve feature recognition. The final feature recognition framework is shown in Figure 1.

#### 2.4.2. 1D-CNN Model Design

**Figure 4:**
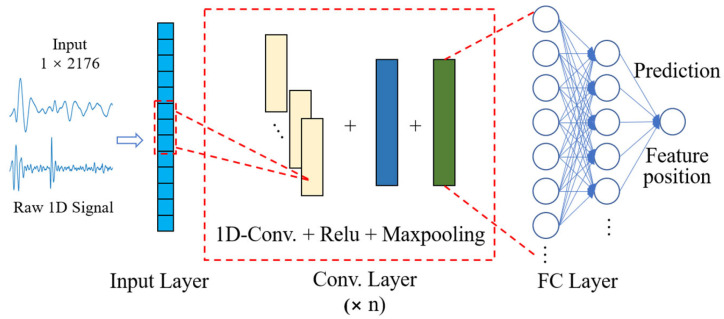
Diagram of the structure of 1D-CNN, including the input layer, convolutional (conv) layer, fully connected (FC) layer, and output layer.

The model we designed is a compact architecture, as shown in Figure 4. The model is mainly composed of four parts: an input layer, convolution layers, fully connected layers, and an output layer. The superimposed convolution layers are used to learn and extract deep features to obtain the location of feature points. Single-period signals of fixed length (zero-padding is performed on signals of insufficient length) are used as the input of the model and are fed to multiple conv layers. Behind each conv layer are a ReLU activation function layer and a Maxpooling layer. The ReLU activation function is used to activate feature layers and increase nonlinear relationships, so that the neural network can complete complex learning tasks and can effectively solve the problem of gradient disappearance and reduce the occurrence of overfitting. The Maxpooling layers are used for downsampling to remove redundant information and enhance output performance. Finally, the filter moves on the time scale to extract and learn time-domain features of vibration signals. In the process of network training, smooth L1 is used as the loss function. The smooth L1 loss function has the advantages of insensitivity to outliers and guaranteeing gradient stability during training, so it is popular in regression problems [37]. Its formula is shown in (4). When the value of smooth L1 loss function does not decrease after 30 epochs, it is considered that the network has been trained.

(4) $$smooth\;L1(x)=\left\{\begin{array}{c} 0.5x^{2},\;\;\;\;if|x|<1\\ |x|-0.5,\;\;\;\;otherwise \end{array}\right.$$

The ten feature points were labeled (eight SCG points were manually labeled and two PCG points were automatically labeled by algorithm), and each feature point was trained separately by the 1D-CNN model. We use Python 3.8 and the Pytorch framework to develop AI models. The model training is performed on NVIDIA 2080 Ti and the operating system is Ubuntu 20.04. During training, the initial learning rate is 0.001 and the batch size is set to 64. The detailed parameters of the model experiment are listed in Table 1.

Based on the processed samples and the trained 1D-CNN model, the model structure is further optimized by optimizing the number of convolutional layers and input–output channels. This results in a more compact model structure with lower latency while maintaining recognition accuracy.

#### 2.4.3. Model Evaluation

The goal of this study is to predict the positional coordinates of the feature points based on the input signal, which belongs to the regression problem in supervised learning. To evaluate the model performance, we use the coefficient of determination $R^{2}$ to reflect model accuracy. This parameter represents the ratio of the regression sum of squares to the residual sum of squares and the closer this statistic is to 1, the better the fit of the model. The root mean square error (RMSE) is used as the training loss and the mean absolute error (MAE) is used as the metric to help determine the model performance. Equations (4)–(6) give the corresponding definitions, where f denotes the location coordinates of the predicted feature points, $\hat{f}$ denotes the predicted value of the feature point coordinates, and $\bar{f}$ denotes the mean value of f. The MAE denotes the sum of the absolute values of the difference between the predicted value and the actual value, which can intuitively reflect the prediction value error. And RMSE is more sensitive to the effect of outliers, which is favorable to the convergence of the model.

(5) $$R^{2}=1-\frac{\sum_{i=1}^{n}{\left(f_{i}-{\hat{f}}_{i}\right)}^{2}}{\sum_{i=1}^{n}{\left(f_{i}-{\bar{f}}_{i}\right)}^{2}}$$

(6) $$MAE=\frac{\sum_{i=1}^{n}\left|f_{i}-{\hat{f}}_{i}\right|}{n}$$

(7) $$RMSE=\sqrt{\frac{\sum_{i=1}^{n}{\left(f_{i}-{\hat{f}}_{i}\right)}^{2}}{n}}$$

## 3. Results

### 3.1. Acquisition System and Signal Preprocessing

**Figure 5:**
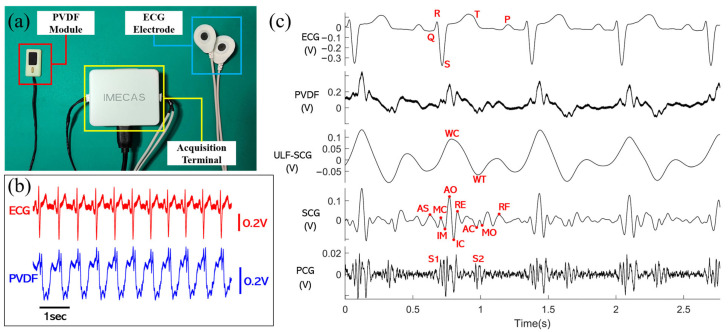
(**a**) The built acquisition system, including the PVDF module, ECG electrodes, and acquisition terminal; (**b**) Raw ECG and raw PVDF signal from one subject with the PVDF attached to the sternum. (**c**) Synchronized signals, from top to bottom, of filtered ECGs, raw PVDF signals, filtered ULF-SCGs, filtered SCGs, and filtered PCGs, with the corresponding feature points labeled.

We built an acquisition system as shown in Figure 5a and acquired synchronized ECG and PVDF vibration signals as shown in Figure 5b. The raw ECG signals were filtered using a zero-phase-shift second-order Butterworth filter with a passband of 1–40 Hz to obtain the filtered ECG signals. The raw PVDF signals were passed through a zero-phase-shift second-order Butterworth filter from 1–5 Hz, 5–30 Hz, and 20–200 Hz to obtain the ULF-SCG, SCG, and PCG signals, respectively, as shown in Figure 5c. The relevant feature points marked in the figure include five classical feature points of ECG (Q, R, S, T, and P), two feature points of ULF-SCG (wave crest WC and wave trough WT) [22], nine standard feature points of SCG [8], and two feature points of PCG. Their related definitions are shown in Table 2.

### 3.2. Data Sources

We selected six volunteers from 23 to 35 years old. Four are males and two are females. During the experiment, all subjects knew the research content well and the recording process. We used our recording prototype to synchronously record the subject’s PVDF and ECG signals. We asked each subject to sit vertically on a chair. After the device was put on and the subject’s breath was smooth, the recording began. After breathing evenly, the subjects held their breath and repeated the procedure twice to thrice for about two minutes. The signals were finally transmitted to and saved on the host computer and about 10-s stable signals in the breath-holding state were selected as valid data for analysis. To cover waveforms with rich morphology and multiple heart rate ranges, we acquired signals from each subject in different states, including calm state and recovery process after daily exercise. The final acquisition yielded 61 multi-cycle data from which 1255 cycles were extracted for model training and testing. The basic physiological parameters of the subjects are shown in Table 3.

### 3.3. Multi-Frequency Vibration Sensor Testing

**Figure 6:**
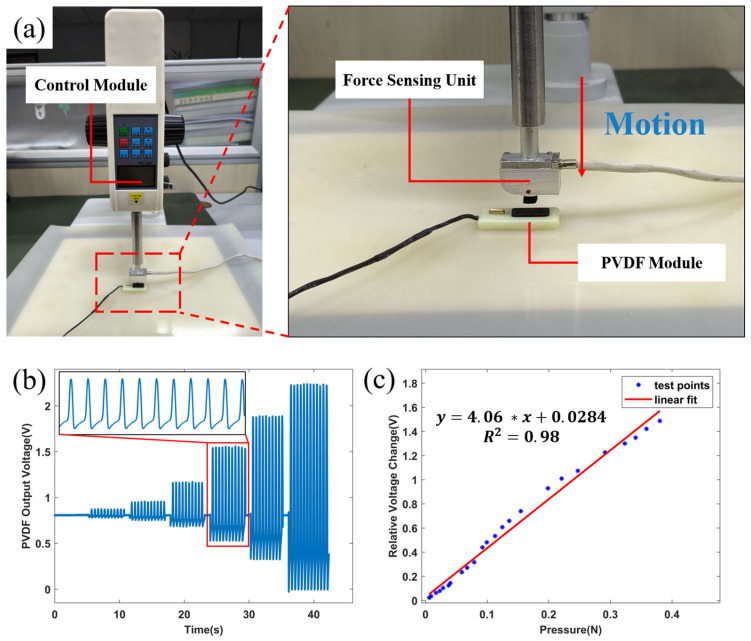
Sensitivity testing of PVDF modules. (**a**) Calibration of the experimental setup, including control module, force sensing unit, and PVDF sensor. (**b**) An experimental procedure with different force levels set to record different voltage outputs. (**c**) Voltage output-force response curve of the PVDF module.

We utilized a calibration experimental setup shown in Figure 6a to calibrate the sensor, where the force-sensing unit moves relative to PVDF periodically. As shown in Figure 6b, the relationship between the output voltage of PVDF and the magnitude of the force is established by the axiom that the action force is equal to the reaction force. As Figure 6c shows, the voltage-force responsive curve is obtained after four calibration experiments. The calculated sensitivity of the multi-frequency sensor module is 40.6 V/N (Tested by reducing the output gain to 1/10 of its original value because the output sensitivity was too high), which satisfies the demand for detecting weak vibration signals of the chest.

**Figure 7:**
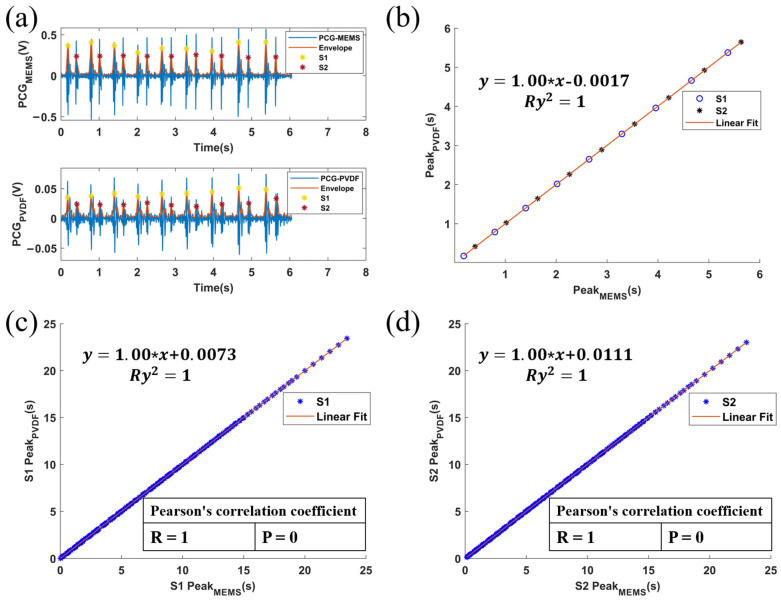
(**a**) Comparison of PCG signals acquired by PVDF and MEMS including the PCG envelope extracted by the algorithm [25] and labeled S1 and S2. (**b**) Correlation of S1 and S2 positions measured by the PVDF module and MEMS (data from (**a**)). (**c**) Correlation of S1 position measured by PVDF module and MEMS (249 signal cycles). (**d**) Correlation of S2 position measured by PVDF module and MEMS (249 signal cycles).

In this study, to verify the PCG collected by our sensor, we selected a microphone sensor WM7120 (Wolfson Company, Edinburgh, UK) to record data synchronously [22]. Figure 7a shows the comparison between two synchronously-recorded PCG signals. Compared with the PCG recorded by the microphone, the PCG recorded by our sensor exhibits almost the same S1 and S2 peaks. We obtained the temporal locations of S1 and S2 by processing the PCG envelope with a previously proposed algorithm [25] and analyzed the correlation of S1 and S2, respectively. The results are shown in Figure 7b–d, where the feature point locations obtained from the two methods have a relatively perfect correlation, verifying that our sensor not only extracts ULF-SCG and SCG but is also effective in extracting PCG. Figure 7c,d are correlation tests performed with 249 cycles of signals acquired through multiple experiments.

### 3.4. Verification of Multi-Frequency Vibration Model

The multi-frequency vibration model of the heart proposed in this study relies on the assumption that the cardiac activity is strictly periodic and linearly stable but the actual chest wall vibration induced by the cardiac activity is a quasi-periodic signal that is non-linear and non-stable. Fourier series and EMD decomposition methods are capable of decomposing time domain signals into different frequency components. The difference is that EMD can decompose non-linear and non-stable signals, which makes it a suitable tool for signal validation. EMD can adaptively decompose the eigenmodes in different frequency ranges from the data, accurately reflecting the dynamics of cardiac vibration signals [38].

**Figure 8:**
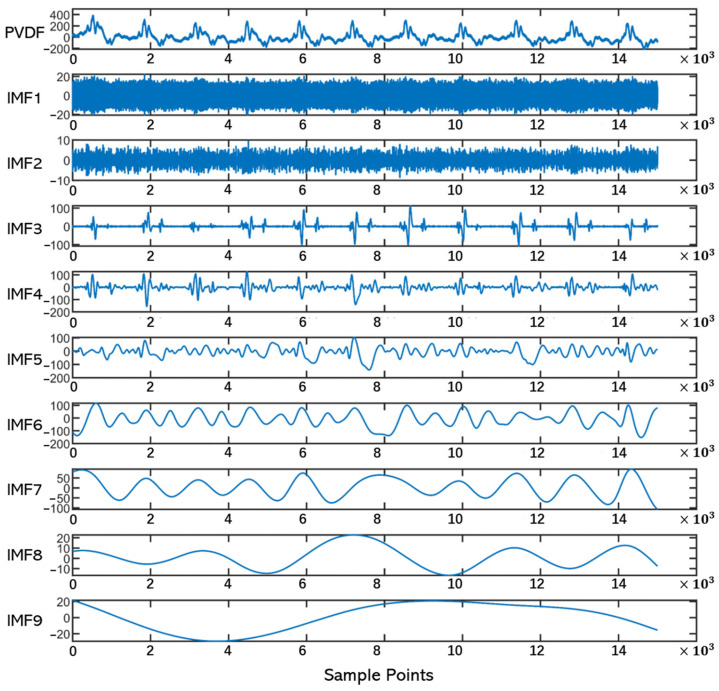
EMD decomposition plots. From top to bottom are the original PVDF waveform and the nine intrinsic modal functions (distributed from high to low frequencies).

**Figure 9:**
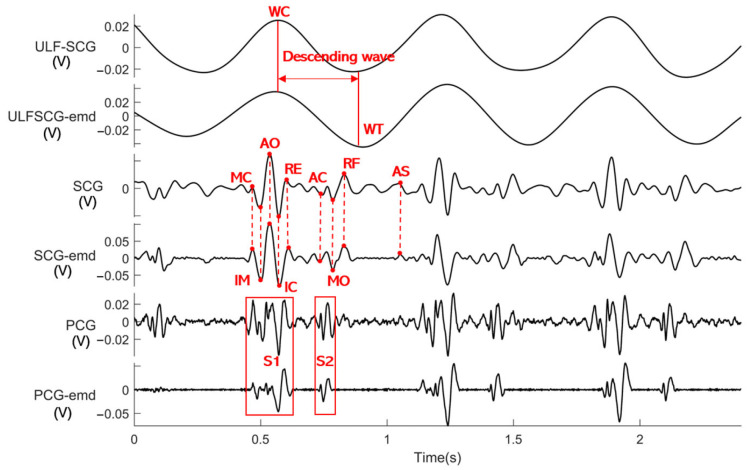
Layer 3, 4, and 7 IMFs are compared with the waveforms and feature points of the actual PCG, SCG, and ULF-SCG, respectively.

We decompose the vibration signal of the PVDF using the EMD method to obtain the multiple intrinsic mode functions IMFs as shown in Figure 8. Each IMF captures the local oscillatory modes of the signal, represents a different frequency component, and generally has a well-defined physical meaning. We find that the IMF functions of layers 3, 4, and 7 correspond to the PCG, SCG, and ULF-SCG, respectively. After comparison, the waveform morphology and feature points are very close to the actual waveforms of the three, as shown in Figure 9. They represent the $f_{n}(t)$ vibrational wave modes described in the vibrational model. Furthermore, we decompose cardiac vibration into three modes as follows:

1. The fundamental frequency mode, represented by ULF-SCG, characterizes the process of changes in cardiac volume caused by systole and diastole;
2. The mid-frequency mode of vibration represented by SCG, which is in the infrasound band and a small number of audible sound bands, and thus contains both low-frequency behaviors, such as rapid diastolic filling peaks and peak atrial contractions, and higher-frequency behaviors, such as valve opening and closing;
3. Higher harmonic modes represented by PCG characterize the high-frequency acoustic information generated by valve opening and closing and blood flow.

In this section, we validate the proposed vibration model through EMD decomposition, where cardiac vibration can be decomposed into multi-frequency vibration modes distinguished by cardiac events. It provides a systematic explanation for the cardiac monitoring of multi-frequency vibration and guides the design of the sensor and signal processing.

### 3.5. Validation of the Feature Extraction Algorithm

#### 3.5.1. Data Set Organization

**Figure 10:**
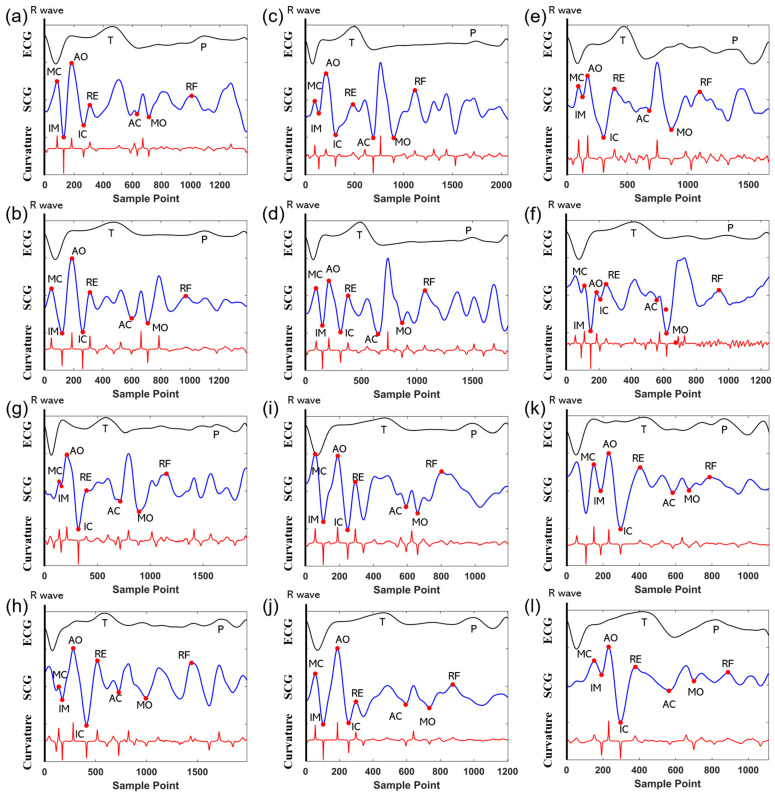
Characteristic point calibration of SCGs with different morphologies, where (**a**–**l**) are two single cycles of data from subjects #1–6, respectively. Where the black, blue, and red curves are the ECG, SCG, and curvature features, respectively. The red dots are the SCG features.

After organizing, 1255 data sets were generated. Each data set includes single-cycle signals of SCG and PCG with a fixed length of 2048 (sampling rate 2 kHz) and includes 10 labeled feature points. The labeling standard and quality of the feature points determine the good or bad results of the model. Because there is no suitable method to accurately label the eight feature points for the SCG signal, we performed the labeling manually and referred to the standard SCG feature point definitions [8]: the MC is the beginning of the sharp drop of the SCG after the start of the QRS wave and the lowest point of the downslope of the MC is labeled as isotonic motion (IM). The peak of the upward-sloping segment from the IM is called the AO point; the isotonic contraction (IC) point is the nadir of the downsloping segment that begins at the AO point. The peak of the circular positive wave after the IC is called the rapid ventricular ejection peak (RE); the AC records the steep-sloping change near the end of the T wave of the ECG (downward); the MO corresponds to the second lowest point of the downslope after the AC; and the peak of the rapid diastolic filling (RF) is identified as the peak of the SCG’s second circular peak after the MO point. Meanwhile, the study [22] illustrated that the curvature near the AO and AC points in the SCG shows a significant peak. Therefore, the synchronized curvature features provide us with an aid in calibrating SCGs of different morphologies. Ultimately, the calibration of the feature points of different morphology SCGs is achieved by strictly following the definition of feature points of SCGs and using the synchronized ECGs and curvature features as an aid. Figure 10 shows the eight feature points calibrated in different morphology SCGs. It can be seen in the figure that there is variability in the signal waveforms between different subjects and there is also variability in the signals of the same subject. The variability may be related to the human body’s weight, height, body fat percentage, muscle strength, gender, and others [39]. Additionally, it may involve the signal propagation and coupling mechanisms of the human heart, chest wall, and sensors at different heart rates. However, we can complete the feature calibration of SCG by feature definition, synchronized ECG, and curvature features.

For the PCG signals, we processed them using a previously proposed algorithm [25], which obtains the locations of stable S1 and S2 by performing K-means clustering and wavelet transforms on the energy envelopes of the heart sounds. The algorithm extracts S1 and S2 with an accuracy of 98.02% and 96.76%, respectively, which can satisfy the high accuracy of automatic extraction. Meanwhile, to ensure the lowest error rate, we visually check the positions of the feature points in the signal waveform during the automatic labeling process and correct the points with errors to maximize the validity of the final results.

#### 3.5.2. 1D-CNN Model Training and Optimization

In this paper, feature point extraction of multi-frequency vibration waves is obtained by 1D-CNN modeling. The deep network models tend to give better training results but they come with an exponential increase in parameters and processing times. Therefore, we often need to make a trade-off between both processing time and recognition accuracy. Generally, the above trade-off can be achieved by adjusting the number of layers of CONV and the number of input and output channels to optimize the model [34].

We varied the number of CONV layers to 3, 4, 5, 6, 7, 8, 9, and 10 for the experiments and the comparative results of $R^{2}$ obtained are shown in Table 4. As can be seen from the table, when the number of convolutional layers is five to six, $R^{2}$ is closest to one, which means that the model predicts best in this range. When the number of convolutional layers is too small, the model is not sufficient to learn useful features and hence the recognition is poor. And when the number of convolutional layers is too large, the model suffers from problems such as overfitting, leading to poor recognition in the test set. In contrast, when the number of convolutional layers is six, the average value of $R^{2}$ is closer to one. Although when the number of convolutional layers is five, the $R^{2}$ of the RF of the feature points is better, the difference is not significant from that of the number of convolutional layers, six. After comprehensive consideration, the number of convolution layers is uniformly set to six.

**Figure 11:**
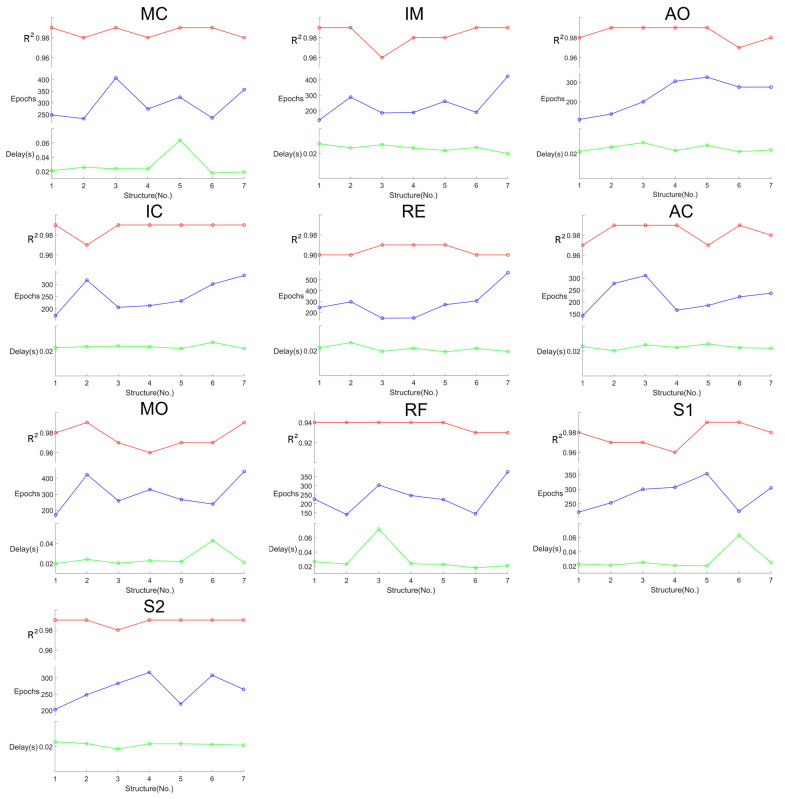
Feature point training results for seven model structures, including $R^{2}$, Epochs under the early stopping rule, and test duration of the model (377 test samples), corresponding to the red, blue and green lines, respectively.

**Figure 12:**
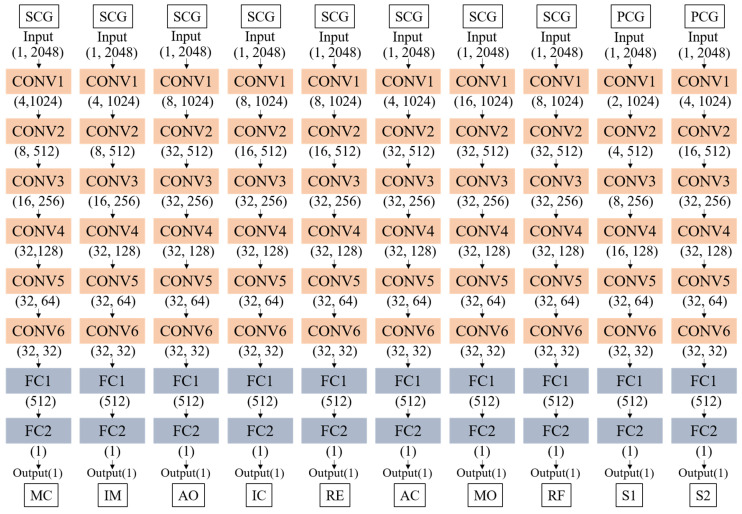
Optimal model structure of ten feature points. MC, IM, AO, IC, RE, AC, MO, RF, S1, and S2 use the sixth, sixth, second, third, third, fourth, first, second, seventh, and fifth model structures in Table 5, respectively.

In order to simplify the model, we modified the structure of the CNN by adjusting the number of input and output channels of each convolutional layer. Based on determining the number of convolutional layers as six, seven network structure models were designed as shown in Table 5. The network includes six convolutional layers and two FC layers. In the structure ‘a-b’ in the table, ‘a’ represents the number of input channels and ‘b’ represents the number of output channels. Different network structures will bring about different network performance and at the same time will lead to a different number of parameters and different latency of the algorithm consequently. In addition, setting the early-stopping rule can effectively avoid entering overfitting due to too many epochs [40]. In this paper, it is set that the network is considered to have been trained when the output value of the loss function of the network does not decrease for 30 epochs. We used 7 different network structures to train the 10 feature points. The obtained model training results are shown in Figure 11. The model training effect ($R^{2}$), algorithm delay (delay), and training epochs are considered to select the network structure adapted to each feature point. Finally, the optimal structural model for the 10 feature points is obtained as shown in Figure 12.

#### 3.5.3. Model Testing and Evaluation

The data of the test dataset of 10 feature points were input into the already trained model and the test results as shown in Table 6 were obtained.

The results of the model training show that the $R^{2}$ values of the feature points MC, IM, AO, IC, AC, and S2 reach 0.99. The $R^{2}$ values of the feature points RE and RF are 0.97 and 0.94, respectively, which are relatively low. We converted the MAE and RMSE parameters to time (original MAE/sample rate, with sample rate equal to 2 kHz) and the MAEs of all feature values were below 2 ms and the RMSEs below 4 ms, except for the points RE and RF. We test the trained model using a test set. The results show that although the $R^{2}$ values of the feature points are all lower than the $R^{2}$ values of the training set, the overall $R^{2}$ values reached 0.9 or above. The MAE of all points except the RF point is below 5 ms; the values of RMSE of all points except RF and RE points are also below 10 ms. Especially, the RMSE of S1, S2, MC, IM, and AO points are below 5 ms. In summary, except for the two points RE and RF, all other feature points were able to achieve high recognition accuracy. The average sizes of $R^{2}$, MAE, and RMSE in the test set reached 0.95, 2.18 ms, and 4.89 ms, respectively. RE and RF represent the peak systolic ejection and diastolic fast-filling peaks, respectively, resulting in less curvature at both points (shown in Figure 10). These two feature points are more gentle waveform features that are less able to cope with disturbances, thus appearing to have lower recognition accuracy and resulting in a different result between the training and testing sets. Figure 10 demonstrates the different morphologies of SCG signals and the above results prove that the 1D-CNN approach can achieve feature extraction with high accuracy for SCG waveforms with different morphologies. We judged that CNN’s method extracted deep signal features for feature point location extraction and the deep signal features may be related to the curvature features [22] because of the variability of curvature features for different feature points. Therefore, we believe the method exhibits strong robustness in extracting features for signals with differences in morphological variability.

Meanwhile, we tested the processing time scale of the 1D-CNN algorithm, as shown in the delay row of Figure 11 and as shown in the test-delay (s) row of Table 6, which demonstrates the length of processing time for all the test set data. The proportion of the test set is 30% of the total dataset, containing a total of 377 samples. The total test time is averaged for each piece of data and the time length of each point obtained is shown in the last row of Table 6. The total average value obtained from the calculation is 60.18 us, which means that the average prediction speed of feature points is 60.18 us/point, which can meet the requirement of real-time processing.

**Figure 13:**
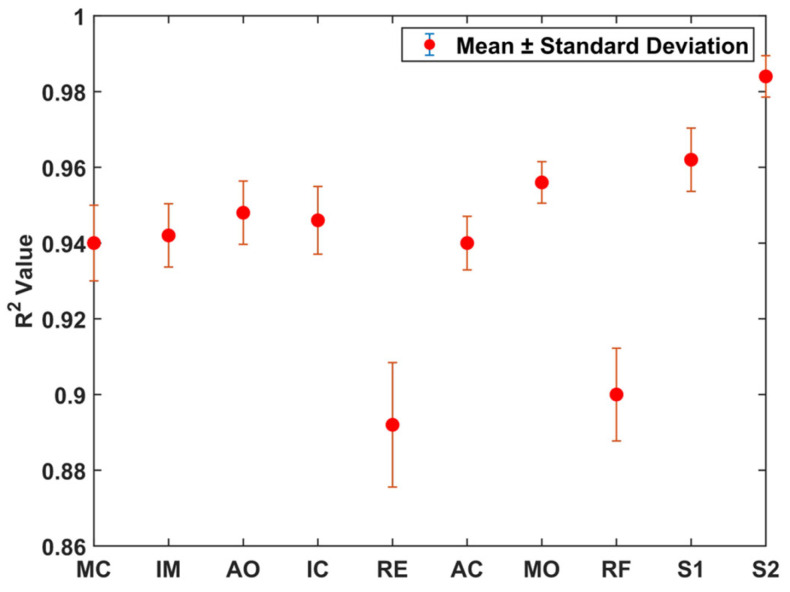
$R^{2}\;$mean and standard deviation results for 10 experiments.

In order to avoid errors introduced by random sample extraction, we randomly divided the samples and repeated the test experiment 10 times. The average $R^{2}$ and standard deviation of each feature point are shown in Figure 13. The mean $R^{2}$ values of each feature were 0.94, 0.94, 0.95, 0.95, 0.89, 0.94, 0.96, 0.9, 0.96, and 0.98, respectively. The mean standard deviation of all points is 0.0091. Except for RE and RF, the mean standard deviation of other points is only 0.0078. It illustrates the high accuracy of the test results.

In addition, to validate the model’s generalization ability for each piece of data, we conducted a 5-fold cross-validation. This involved dividing the dataset into five parts, with one as the test set and the remaining four as the training set for five training and testing sessions. The experimental results are shown in Table 7. Among them, the mean value of $R^{2}$ of five experiments is close to the test results in Table 6. Except for the RE and RF, where the recognition accuracy drops significantly in a few data, the results of the other points do not fluctuate much overall, which shows that our model has good generalization performance.

In summary, first of all, our model faces challenges in recognizing the subtle heart features. RE and RF are more susceptible to noise interference because their curvature features are significantly smaller than the other feature points (shown in Figure 10). It leads to a decrease in the accuracy of the model in recognizing the two, as well as a significant difference between the results of RE and RF in the training set and the test set. However, our model achieves high recognition accuracy for other feature points with low latency. The method can perform feature extraction for waveforms of different morphologies with high robustness and exhibit good generalization performance.

## 4. Discussion

This study first proposes a multi-frequency vibration model of the heart and designs the corresponding sensor and processing algorithms. The vibration model, the sensor, and feature extraction algorithms are integrated to form a multi-frequency vibration monitoring system for the heart. The system expands the frequency range of cardiac monitoring to include the ultra-low frequency band, infrasound band, and audible band, which can extract the comprehensive mechanical vibration information of the heart and combine it with the ECG to map more comprehensive cardiac information.

In our previous study, an electro-mechanical-acoustic activity model of the heart was proposed, emphasizing the association between electrical activity, low-frequency vibration, and high-frequency acoustic activity. Among them, low-frequency vibration and high-frequency acoustic activity are mechanically coupled as the system command response and subsequent higher harmonics of the heart pump, respectively [22]. In this study, we further enrich the connotation of mechanical coupling and elaborate a theoretical model of multi-frequency vibration of the heart in combination with Fourier series theory. It is a new attempt to decompose the quasi-periodic vibration of the heart into vibration modes of different frequency band ranges according to the cardiac events: the fundamental frequency mode represented by ULF-SCG, the mid-frequency vibration mode represented by SCG, and the high-harmonic mode represented by PCG.

A multi-frequency vibration monitoring system for the heart is built based on vibration modeling, including sensor design and feature extraction algorithms. The sensors designed in this study can detect multi-frequency vibration signals compared to existing accelerometers, gyroscopes, etc. A comparison of multi-band vibration sensors is shown in Table 8. Compared to existing sensors [23,24], it is more miniaturized and lightweight while satisfying high sensitivity. Compared to our previous study [22], the sensor in this study becomes enhanced and optimized as the separate sensor structure is more compatible with the wearable design and the static pressure sensor is removed to obtain a smaller size. The 1D-CNN-based feature extraction framework proposed in this study can achieve high-accuracy extraction of multiple vibration feature points, including eight feature points of SCG and two feature points of PCG. A comparison of the feature extraction methods for SCG is shown in Table 9. The method can recognize more feature points compared with the existing Gaussian mixture model [26], high-frequency envelope [27], and wavelet transform [28]. Compared to the methods of binary classification [29], it can ignore the morphological variability of the signal. Compared to the curvature method [22], it can track the feature points of continuous signals and satisfy the low latency property, which can be applied to real-time monitoring.

The cardiac signals measured by the multi-frequency monitoring system are subject to motion artifacts and other disturbances, so we ensured that the subject remained stationary during the acquisition and applied a band-pass filter in the signal processing. It eliminates motion artifacts at low frequencies and noise at high frequencies to some extent for the current acquisition. Current applications are limited to daily stationary continuous monitoring. If we can eliminate interference during movement, we can expand the application to most everyday states, including active states such as walking and climbing stairs. Multi-channel detection would be potentially an effective way to eliminate motion interference [11], where comparing signals from different channels and utilizing signal processing methods can eliminate the effects of motion interference. The sensor designed in this study meets the miniaturization characteristics and can meet the requirements of wearable morphology but, at present, the wearable functions of our devices have not been realized. To meet the requirement of being wearable, we need to solve the problems of wireless transmission, acquisition circuit integration, and power supply and at the same time realize the low power consumption and miniaturization design. For the 1D-CNN model, the current sample size of 1255 cannot cover all morphologies of waveforms, which will lead to a decrease in the recognition accuracy when confronted with new morphologies of waveforms. We can solve this problem gradually by increasing the sample size and waveform morphology, such as extensive data collection for healthy and non-healthy populations. In addition, the problem of insufficient sample size can be solved by increasing data diversity through data augmentation. It is also worth noting that the latency time test of the 1D-CNN model was performed on the above GPU computing platform. The delay time of the test includes model prediction and performance evaluation, excluding the time of data loading, and finally obtains an average prediction time of 60.18 us. Compared with the study of the same 1D-CNN method for estimating stress, the prediction time is 115.5 us/sample [41], which can prove the low latency property of our proposed method. Meanwhile, the latency of algorithms running on embedded platforms changes because of the difference in arithmetic power. FPGA platforms are a good choice for embedded platforms because they can provide parallel computing. The compact CNN model clipped in this study can be implemented on FPGA and further performance enhancement can be conducted by computational kernel optimization, broadband optimization, etc. [42,43]. Our study demonstrates that the 1D-CNN method can obtain high-accuracy recognition results while satisfying the low latency property. Therefore, the latency test at this stage is reasonable.

## 5. Conclusions

This paper proposes a model of multi-frequency vibration to provide a systematic explanation of multi-frequency vibration detection of the heart. The monitoring system constructed based on this model achieves simultaneous detection of ULF-SCG, SCG, and PCG on a single sensor. A feature extraction algorithm framework is also designed based on the 1D-CNN model. We experimentally obtain an efficient recognition model that meets the low latency while ensuring high recognition accuracy. By reducing the sensor size and improving the algorithm efficiency, we finally achieved the goals of portability and low latency.

In the future, we will further optimize the functionality of our system to reach the goal of wearable forms and fully automated monitoring to expand the application scenarios. We will apply the system to extensively and continuously monitor healthy people and cardiac patients and expand the sample size to optimize our algorithmic model. We believe that the system will provide meaningful data and models for dynamic monitoring of heart disease and be able to effectively predict the early development of heart disease.

## Figures and Tables

**Table 1:** Experimental detailed parameters of the 1D-CNN model.

Parameter	Value
Learning	0.0001
Batch Size	64
Epochs	1000
Patience	30
Kernel Size	3 × 1
Pooling Size	2 × 1
Stride	2
Loss Function	Smooth L1
Activation Function	ReLu
AI Framework	Python 3.8, Pytorch
GPU	NVIDIA 2080 Ti
Operating System	Ubuntu 20.04

**Table 2:** Definition of signal feature points.

Signal	Feature	Definition
ULF-SCG	WC	WC-WT: cardiac ejection period
	WT	
SCG	AS	Peak of atrial systole
	MC	Mitral valve closure
	IM	Isovolumic movement
	AO	Aortic valve opening
	IC	Isobaric contraction
	RE	Peak of rapid systolic ejection
	AC	Aortic valve closure
	MO	Mitral valve opening
	RF	Peak of rapid diastolic filling
PCG	S1	Mitral and tricuspid valves closure
	S2	Aortic and pulmonary valves closure

**Table 3:** Relevant information on the six subjects counted.

Variables	Value
Gender-Male	4
Gender-Female	2
Age (year)	28.3 ± 3.8 (23–35)
Height (cm)	171.2 ± 8.9 (160–188)
Weight (Kg)	62.5 ± 11.6 (52–83)
BMI	21.3 ± 2.9 (17.6–26.493)

**Table 4:** The $R^{2}$ results for different numbers of convolutional layers.

Feature	Conv3	Conv4	Conv5	Conv6	Conv7	Conv8	Conv9	Conv10
MC	−7.43	−7.38	−7.85	0.99	0.98	0.98	0.89	−3.51
IM	−18.79	−17.76	0.97	0.99	0.97	0.97	−0.3	−10.75
AO	−30.74	−28.18	0.98	0.99	0.99	0.97	−3.43	−20.12
IC	−35.29	−39.68	0.97	0.99	0.99	−0.26	−11.15	−28.15
RE	0.89	0.92	0.96	0.97	0.94	−2.84	−13.95	−28.17
AC	−121.86	−34.87	0.97	0.98	−3.46	−42.61	−75.09	−110.21
MO	−64.75	−7.58	0.98	0.99	−6.21	−28.66	−44.41	−61.28
RF	0.96	0.98	0.97	0.94	−10.26	−29.78	−43.27	−59.12
S1	−25.25	−25.7	−26.54	0.98	0.99	0.99	−0.18	−7.54
S2	−122.86	−125.08	0.99	0.99	−4.33	−43.7	−78.41	−99.84

**Table 5:** The seven model structures.

No.	Layer 1	Layer 2	Layer 3	Layer 4	Layer 5	Layer 6	Layer 7–8 (FC)
1	1–16	32–32	32–32	32–32	32–32	32–32	1024–512, 512–1
2	1–8	8–32	32–32	32–32	32–32	32–32	
3	1–8	8–16	16–32	32–32	32–32	32–32	
4	1–4	4–32	32–32	32–32	32–32	32–32	
5	1–4	4–16	16–32	32–32	32–32	32–32	
6	1–4	4–8	8–16	16–32	32–32	32–32	
7	1–2	2–4	4–8	8–16	16–32	32–32	

**Table 6:** Model test results.

Signal		SCG								PCG	
Feature		MC	IM	AO	IC	RE	AC	MO	RF	S1	S2
Structure		Layer 6	Layer 6	Layer 2	Layer 3	Layer 3	Layer 4	Layer 2	Layer 2	Layer 1	Layer 5
Train	$R^{2}$	0.99	0.99	0.99	0.99	0.97	0.99	0.98	0.94	0.98	0.99
	MAE (ms)	0.585	0.835	0.845	0.86	3.365	1.42	1.38	6.035	0.84	0.575
	RMSE (ms)	0.805	1.445	1.295	1.565	7.28	3.32	3.81	13.42	1.08	0.885
Test	$R^{2}$	0.95	0.94	0.95	0.94	0.90	0.94	0.96	0.91	0.97	0.98
	MAE (ms)	2.145	1.975	1.75	1.73	4.425	3.035	4.345	8.32	1.21	1.235
	RMSE (ms)	4.245	4.205	3.85	5.48	10.415	7.055	9.63	16.315	2.265	2.425
Test	Delay (s)	0.0179	0.02485	0.02535	0.02394	0.0196	0.02289	0.0241	0.02308	0.02294	0.02224
	Delay (us/Point)	47.48	65.92	67.24	63.5	51.99	60.72	63.93	61.22	60.85	58.99

**Table 7:** $R^{2}$ results for 5-fold cross-validation.

Feature	No. 1	No. 2	No. 3	No. 4	No. 5	Average	Standard Deviation
MC	0.94	0.94	0.95	0.96	0.94	0.946	0.00894
IM	0.96	0.95	0.94	0.96	0.94	0.950	0.01
AO	0.96	0.96	0.94	0.95	0.96	0.954	0.00894
IC	0.95	0.97	0.96	0.95	0.94	0.954	0.01140
RE	0.88	0.91	0.89	0.94	0.87	0.898	0.02774
AC	0.97	0.96	0.96	0.95	0.95	0.958	0.00836
MO	0.98	0.98	0.97	0.98	0.97	0.976	0.00547
RF	0.91	0.92	0.89	0.9	0.88	0.900	0.01581
S1	0.98	0.98	0.98	0.97	0.96	0.974	0.00894
S2	0.99	0.99	0.98	0.99	0.99	0.988	0.00447

**Table 8:** Comparisons with other multi-band signal sensor modules.

Sensor Module	3D Size (mm)	Volume (mm^3^)	Weight (g)	Extracted Signals	Power Supply
CM-01B [24]	Ø20 (diameter) × 10	3140	4.3	SCG, PCG	Yes
PZT [23]	Ø30 (diameter) × 10	7065	Not mentioned	LF-FCG, HF-FCG, HS-FCG	No
Composite Sensor [22]	Designed on an integrated device.			ULF-SCG, SCG	No
Proposed	30 × 15 × 5	2250	2.4	ULF-SCG, SCG, PCG	No

**Table 9:** Comparisons with other feature extraction methods.

Feature Extraction Method	Identified Feature Points	Morphological Variability Robustness	Meet Low Latency
Gaussian mixture model [26]	AO, AC	Poor	Yes
High-frequency envelope [27]	AC, IM	Robust	No
Wavelet transform [28]	AO, IM	Poor	No
Binary classification [29]	AS, MC, IM, AO, IC, RE, AC, MO, RF	Poor	No
Curvature method [22]	AS, MC, IM, AO, IC, RE, AC, MO, RF	Robust	No
Proposed	MC, IM, AO, IC, RE, AC, MO, RF	Robust	Yes

## Data Availability

The raw/processed data required to reproduce these findings cannot be shared at this time as the data also form part of an ongoing study.
